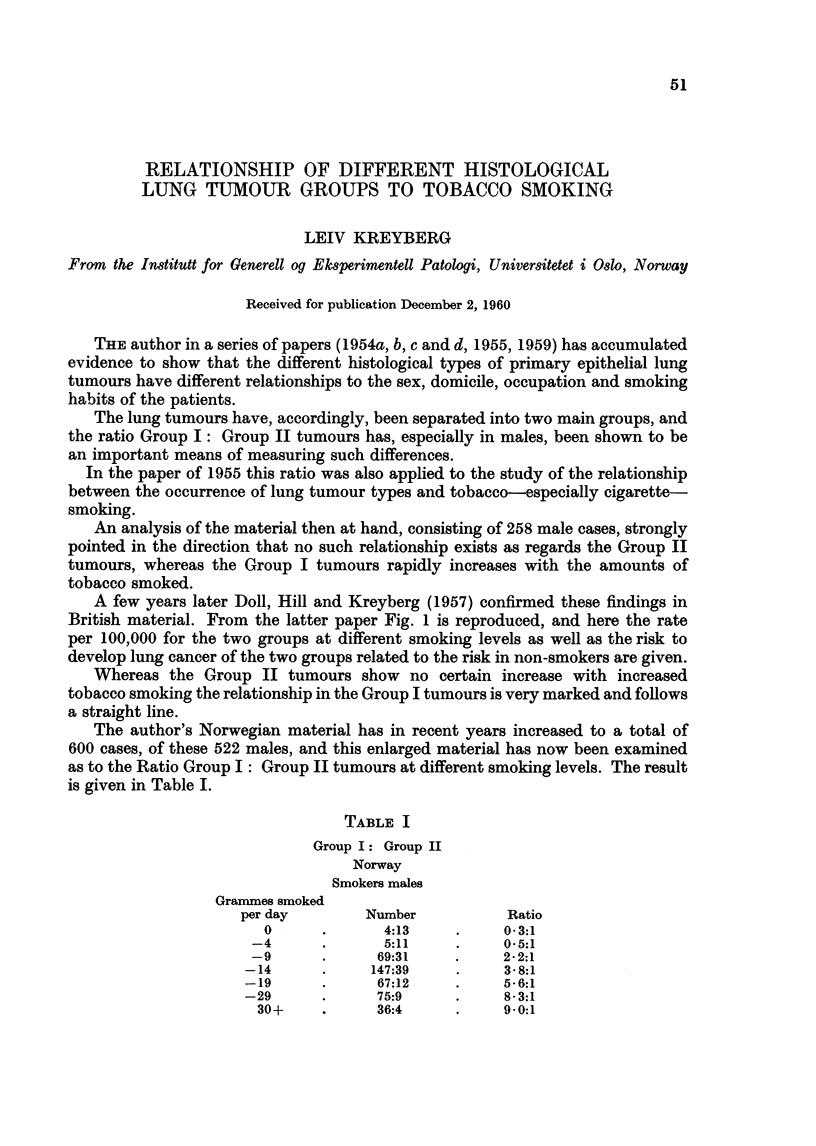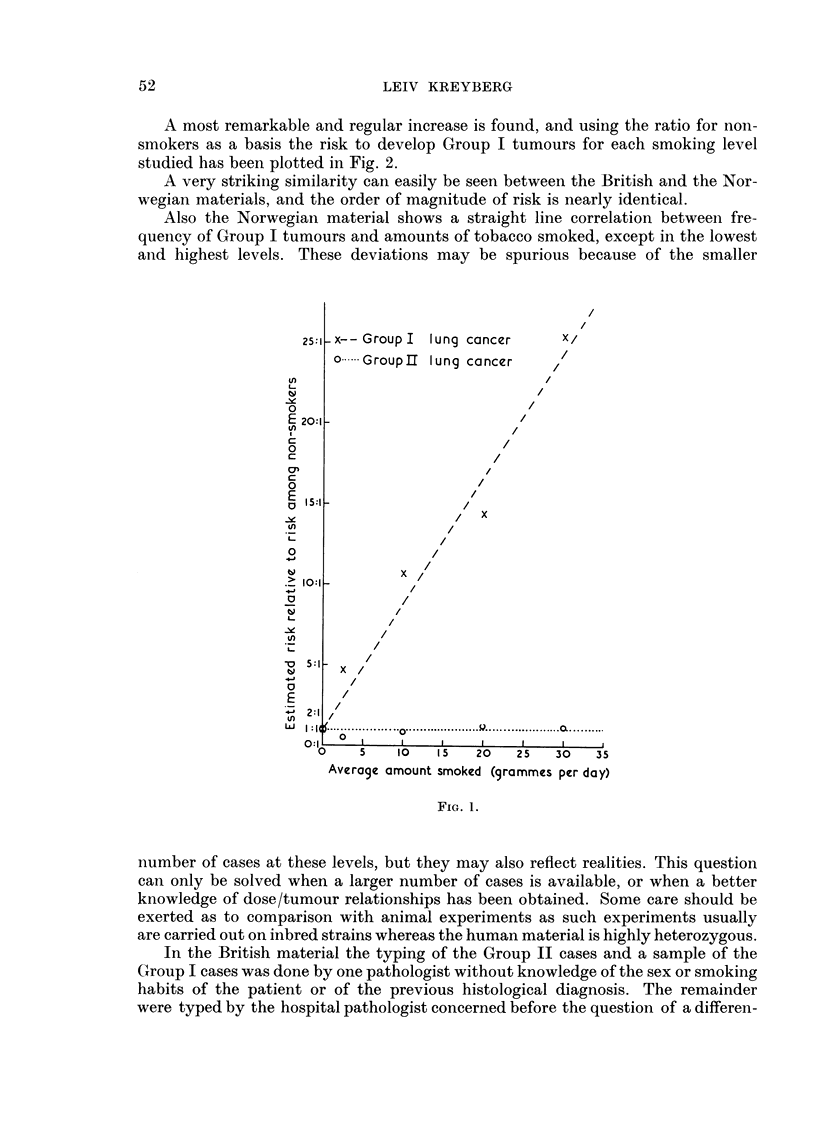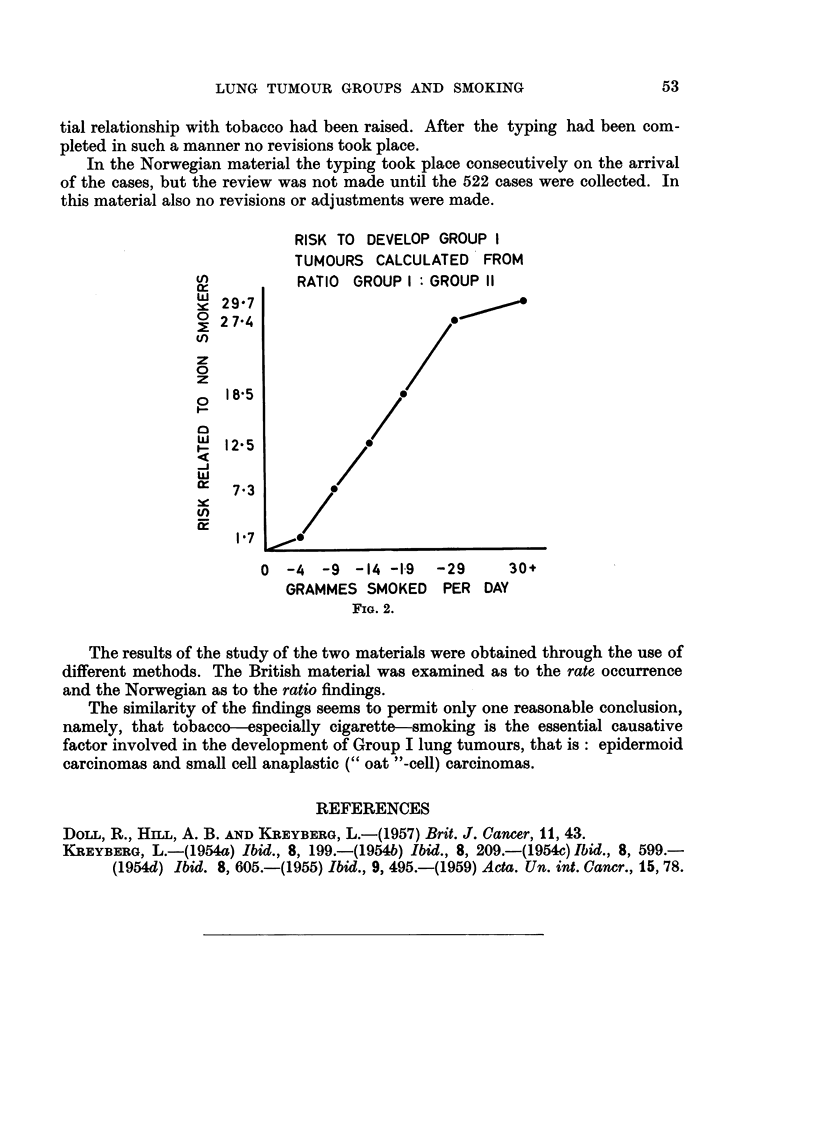# Relationship of Different Histological Lung Tumour Groups to Tobacco Smoking

**DOI:** 10.1038/bjc.1961.7

**Published:** 1961-03

**Authors:** Leiv Kreyberg


### RELATIONSHIP OF DIFFERENT HISTOLOGICAL LUNG TUMOUR GROUPS TO TOBACCO SMOKING

LEIV KREYBERG

*From the Institutt for Generell og Eksperimentell Patologi, Universitetet i Oslo, Norway*

Received for publication December 2, 1960

THE author in a series of papers (1954*a*, *b*, *c* and *d*, 1955, 1959) has accumulated evidence to show that the different histological types of primary epithelial lung tumours have different relationships to the sex, domicile, occupation and smoking habits of the patients.

The lung tumours have, accordingly, been separated into two main groups, and the ratio Group I : Group II tumours has, especially in males, been shown to be an important means of measuring such differences.

In the paper of 1955 this ratio was also applied to the study of the relationship between the occurrence of lung tumour types and tobacco—especially cigarette—smoking.

An analysis of the material then at hand, consisting of 258 male cases, strongly pointed in the direction that no such relationship exists as regards the Group II tumours, whereas the Group I tumours rapidly increases with the amounts of tobacco smoked.

A few years later Doll, Hill and Kreyberg (1957) confirmed these findings in British material. From the latter paper Fig. 1 is reproduced, and here the rate per 100,000 for the two groups at different smoking levels as well as the risk to develop lung cancer of the two groups related to the risk in non-smokers are given.

Whereas the Group II tumours show no certain increase with increased tobacco smoking the relationship in the Group I tumours is very marked and follows a straight line.

The author's Norwegian material has in recent years increased to a total of 600 cases, of these 522 males, and this enlarged material has now been examined as to the Ratio Group I : Group II tumours at different smoking levels. The result is given in Table I.

TABLE I

Group I : Group II
Norway
Smokers males

| Grammes smoked per day | Number | Ratio |
|---|---|---|
| 0 | 4:13 | 0·3:1 |
| –4 | 5:11 | 0·5:1 |
| –9 | 69:31 | 2·2:1 |
| –14 | 147:39 | 3·8:1 |
| –19 | 67:12 | 5·6:1 |
| –29 | 75:9 | 8·3:1 |
| 30+ | 36:4 | 9·0:1 |

A most remarkable and regular increase is found, and using the ratio for non-smokers as a basis the risk to develop Group I tumours for each smoking level studied has been plotted in Fig. 2.

A very striking similarity can easily be seen between the British and the Norwegian materials, and the order of magnitude of risk is nearly identical.

Also the Norwegian material shows a straight line correlation between frequency of Group I tumours and amounts of tobacco smoked, except in the lowest and highest levels. These deviations may be spurious because of the smaller

FIG. 1.

number of cases at these levels, but they may also reflect realities. This question can only be solved when a larger number of cases is available, or when a better knowledge of dose/tumour relationships has been obtained. Some care should be exerted as to comparison with animal experiments as such experiments usually are carried out on inbred strains whereas the human material is highly heterozygous.

In the British material the typing of the Group II cases and a sample of the Group I cases was done by one pathologist without knowledge of the sex or smoking habits of the patient or of the previous histological diagnosis. The remainder were typed by the hospital pathologist concerned before the question of a differential relationship with tobacco had been raised. After the typing had been completed in such a manner no revisions took place.

In the Norwegian material the typing took place consecutively on the arrival of the cases, but the review was not made until the 522 cases were collected. In this material also no revisions or adjustments were made.

FIG. 2.

The results of the study of the two materials were obtained through the use of different methods. The British material was examined as to the *rate* occurrence and the Norwegian as to the *ratio* findings.

The similarity of the findings seems to permit only one reasonable conclusion, namely, that tobacco—especially cigarette—smoking is the essential causative factor involved in the development of Group I lung tumours, that is : epidermoid carcinomas and small cell anaplastic (" oat "-cell) carcinomas.

### REFERENCES

DOLL, R., HILL, A. B. AND KREYBERG, L.—(1957) *Brit. J. Cancer*, **11**, 43.
KREYBERG, L.—(1954*a*) *Ibid.*, **8**, 199.—(1954*b*) *Ibid.*, **8**, 209.—(1954*c*) *Ibid.*, **8**, 599.—(1954*d*) *Ibid.* **8**, 605.—(1955) *Ibid.*, **9**, 495.—(1959) *Acta. Un. int. Cancr.*, **15**, 78.